# Toddalolactone protects against osteoarthritis by ameliorating chondrocyte inflammation and suppressing osteoclastogenesis

**DOI:** 10.1186/s13020-022-00576-w

**Published:** 2022-02-05

**Authors:** Yiming Xu, Song Xue, Tian Zhang, Xinmeng Jin, Cong Wang, Haiming Lu, Yiming Zhong, Hongjie Chen, Libo Zhu, Jinzhong Ma, Weilin Sang

**Affiliations:** 1 Department of Orthopedics, Shanghai General Hospital, Shanghai Jiao Tong University School of Medicine, Shanghai, China; 2 Shanghai Bone Tumor Institution, Shanghai, China; 3 Department of Rheumatology and Immunology, Arthritis Research Institute, The First Affiliated Hospital of Anhui Medical University, Hefei, China; 4 Department of Orthopedics, The Affiliated Yueqing Hospital, Wenzhou Medical University, Wenzhou, China

**Keywords:** Toddalolactone, Cartilage, Inflammation, Osteoclastogenesis, Osteoarthritis

## Abstract

**Background:**

Osteoarthritis (OA) is widely recognized as the most common chronic joint disease accompanied by progressive cartilage and subchondral bone damage. Toddalolactone (TOD), a natural compound extracted from *Toddalia asiatica* (L.) Lam., has been widely used in the treatment of stroke, rheumatoid arthritis, and oedema. Nevertheless, what TOD acts as in the pathogenesis and progression of OA hasn’t been reported. In this investigation, we have aimed to determine how TOD affects OA in vitro and in vivo.

**Methods:**

LPS (10 µg/ml) and IL-1β (10 ng/ml) were employed to induce chondrocyte inflammation or RANKL to induce osteoclast differentiation in bone marrow derived macrophages (BMMs). The effects of TOD on chondrocyte inflammation and osteoclast differentiation were evaluated. Anterior cruciate ligament transection (ACLT) was performed to develop an OA animal model and study the effects of TOD.

**Results:**

We found that TOD inhibited the expression of inflammatory and catabolic mediators (IL-6, IL-8, TNF-α, MMP2, MMP9, and MMP13) in inflammatory chondrocytes in vitro. Furthermore, TOD was proven to inhibit RANKL-induced-osteoclastogenesis and inhibit the expression of osteoclast marker genes. Our data also confirmed that TOD suppressed the destruction of articular cartilage and osteoclastogenesis via inhibiting the activation of NF-κB and MAPK signalling pathways. In the ACLT mouse model, we found that TOD attenuated cartilage erosion and inhibited bone resorption.

**Conclusions:**

These results showed that TOD can be adopted as a potential therapeutic agent for OA.

**Supplementary Information:**

The online version contains supplementary material available at 10.1186/s13020-022-00576-w.

## Background

Osteoarthritis (OA) is widely recognized as the most common chronic joint disease [1]. As the population ages and with obesity on the rise, the incidence of OA is increasing. According to the Rotterdam study [2], 53% of women and 33% of men over the age of 80 had OA of the knee. Osteoarthritis often causes the destruction of articular cartilage and subchondral bone sclerosis, resulting in inflammation and subsequent stiffness, swelling, and difficulty in moving the joint [3, 4]. Pain and loss of function seriously reduce patients’ quality of life, and the high cost of treatment greatly increases the social and economic burden [5]. Considering the complex pathogenesis and high incidence of OA, it is necessary to explore more treatment methods.

Osteoarthritis was thought to be just a “wear and tear” disease [6]. Currently, our understanding of OA has changed dramatically. Osteoarthritis can have a significant impact on articular cartilage, leading to serious degeneration of articular cartilage in the course of the disease, which often leads to low-grade inflammatory features of the cartilage [7, 8]. After inflammatory stimulation, the chondrocytes transform into a degraded phenotype, which not only activates nuclear factor-kappaB (NF-κB) and mitogen-activated protein kinase (MAPK) signalling pathways but also leads to the secretion of a series of inflammatory factors, comprising matrix metalloproteinases (MMPs) and a disintegrin and metalloproteinases with thrombospondin motifs (ADAMTS) [9–11]. Osteoarthritis can gradually lead to cellular changes, structural defects, and dysfunction of the entire interarticular compartment [12, 13]. It is defined as a whole-joint disease, which causes the rupture of articular cartilage and causes subchondral sclerosis [9, 14]. The subchondral bone is located at the epiphyseal layer below the articular cartilage [15]. Bone homeostasis depends on osteoblast-mediated bone formation and osteoclast-mediated bone resorption, especially osteoclast-mediated bone resorption [16, 17]. Osteoclasts originate from the monocyte/macrophage hematopoietic system. During osteoclast survival, differentiation, and maturation, macrophage colony stimulating factor (M-CSF) and RANKL play a key role [18]. Specifically, M-CSF binds to the osteoclast receptor c-Fms to promote the survival and proliferation of osteoclasts, and RANKL binds to the osteoclast surface receptor RANK to promote osteoclast differentiation [19]. The combination of RANKL and RANK recruits tumor necrosis factor receptor-associated factor 6 (TRAF6), which starts a series of cellular signalling cascades, including NF-κB, and mitogen-activated protein kinases (MAPKs, including ERK, JNK and p38) pathways [20]. The cascades eventually lead to the activation and accumulation of nuclear factor of activated T cells c1 (NFATc1) and c-fos, which are core transcriptional factors in the differentiation and maturation of osteoclasts. Then NFATc1 directly regulates the expression of osteoclast-related genes, including tartrate-resistant acid phosphatase (TRAP), cathepsin K (CTSK), dendritic cell-specific transmembrane protein (DC-STAMP), calcitonin receptor (CTR), and VATPase D2, which are responsible for the function of osteoclasts and bone resorption [19, 21]. Therefore, inhibition of osteoclast formation and maintain the balance between these two processes are important for subchondral bone stability during OA [22, 23]. Disruption of joint homeostasis often leads to excessive activation of osteoclasts, causing increased bone resorption and subchondral densification, thereby contributing to the progression of OA [24]. Therefore, it is of great significance to find drugs that can prevent cartilage degeneration and activate osteoclasts for the treatment of OA.

It has been considered that chondrosarcoma SW1353 cells are suitable cellular model for OA research [25–27]. LPS and IL-1β stimulation result in a decrease in type II collagen and aggrecan levels, which are the two main organic components of articular cartilage [9]. This demonstrates that SW1353 cells have chondrocyte cytological characteristics. Additionally, RAW264.7 exhibits biological behavior similar with that of the osteoclast precursor, which can be transformed to osteoclasts by appropriate cytokine stimulation [28, 29]. These two cell types are easy to culture and grow rapidly and thus are used to study the potential anti-chondrocyte inflammation and anti-osteoclastogenesis effects of traditional Chinese medicines. Over the past few decades, compounds extracted from traditional Chinese medicinal plants show strong anti-inflammatory effects and may hold the key to treating OA [19, 30]. Toddalolactone (TOD) is a natural compound extracted from *Toddalia asiatica* (L.) Lam., and has long been used for treating various chronic diseases [31]. A previous study reported that TOD has anti-embolism, hemostatic and anti-fibrotic effects [32]. TOD is a traditional Chinese medicine distributed in Guangxi, Yunnan, and Guizhou provinces, and is broadly adopted for treating diseases like stroke, rheumatoid arthritis, and oedema [33]. Nevertheless, what TOD acts as in OA pathogenesis and progression has not yet been reported.

In the investigation, we have investigated how TOD affects anti-chondrocyte inflammation and inhibition of osteoclastogenesis, and further explored the possibility of TOD as a new potential drug for OA.

## Materials and approaches

### Reagents and cell lines

Toddalolactone (MF: C16H20O6, MW:308.33) came from MedChem Express (Shanghai, China). Dimethyl sulfoxide (DMSO), Penicillin, streptomycin, and tartrate-resistant acid phosphate (TRAP) came from Sigma-Aldrich (USA). RIPA lysis buffer came from Beyotime (Shanghai). Lipopolysaccharides (LPS) and macrophage colony stimulating factor (M-CSF) came from Peprotech (USA). RANKL came from R&D Systems (USA). Primary bone marrow-derived macrophages (BMMs) were separated from the bone marrow of 5-week-old male C57BL/6 mice. SW1353 and RAW264.7 cell lines came from the Cell Bank of Chinese Academy of Sciences (Shanghai).

### Cell viability

The cell counting Kit-8 (CCK-8) analysis (Dojindo, Japan) was employed for assessing TOD toxicity. SW1353, BMMs, and RAW264.7 cells were cultured in 96-well plates with a density of 7 × 10^3^ cells/well. After pre-treatment with gradient concentrations (0, 0.5, 1, 5, 10, 20, or 40 µM) of TOD for the indicated time or being left without treatment, 10 µl CCK-8 was added to the 96-well plate, followed by a 3 h incubation at 37 °C. The optical density (OD) at 450 nm was measured with a microplate photometer.

### Flow cytometry analysis

SW1353 cells were inoculated on 6-well plates (3.0 × 10^5^/well) and treated with gradient concentrations of TOD (0, 0.5, 1, 10, 20, or 40 µM) for 24 h. Cells were gathered after treatment and stained with Annexin V-PE and 7-AAD assay kits on basis of the manufacturer’s instructions.

### Western blotting

After treatment under different conditions, total proteins were collected from the cells adopting ice-cold RIPA lysis buffer. BCA protein assay (Beyotime, China) were adopted for measuring relative protein concentrations. After separation on SDS-PAGE gels, proteins were transferred to the PVDF membrane and sealed with 5% skimmed milk for 2 h. Then the membrane was incubated overnight with a first antibody at 4 °C. Enhanced chemiluminescence was employed for visualizing the bands. The following antibodies were used: PARP (9532, Cell Signalling Technology), C-PARP (5625, Cell Signalling Technology), Bcl-xl (2764, Cell Signalling Technology), Bcl-2 (3498, Cell Signalling Technology), Bax (5023, Cell Signalling Technology), β-Tubulin (10094-1-AP, Proteintech), Trap (ab52750, Abcam), Ctsk (ab187647, Abcam), c-fos (Ab222699, Abcam), NFATc1 (Ab253477, Abcam), GAPDH (60,004–1-lg, Proteintech), P-P65 (3033, Cell Signalling Technology), P65 (8242, Cell Signalling Technology), IκBα (4814, Cell Signalling Technology), P-ERK (4370, Cell Signalling Technology), ERK (4695, Cell Signalling Technology), P-P38 (4511, Cell Signalling Technology), P38 (8690, Cell Signalling Technology), P-JNK (9255, Cell Signalling Technology), JNK (9252, Cell Signalling Technology), and H3 (17168-1-AP, Proteintech).

### Real-time PCR analysis

After a specific treatment, total mRNA was extracted from various cells with TRIZOL then reverse transcribed into cDNA adopting PrimeScript RT Master Mix (TaKaRa, Dalian, China). Real-time PCR was performed with TB Green Premix Ex Tap on basis of the manufacturer’s instructions. The primers employed are listed below: IL-6, forward 5′-CAACCTGAACCTTCCAAAGATG-3′, reverse 5′-ACCTCAAACTCCAAAAGACCAG-3′; IL-8, forward 5′-GAGAGTGATTGAGAGTGGACCAC-3′, reverse 5′-CACAACCCTCTGCACCCAGTTT-3′; TNF-α, forward 5′-CACTTCGAAACCTGGGATTCAG-3′, reverse 5′-GGTCTCCAGATTCCAGATGTCAG-3′; MMP2, forward 5′-AGCGAGTGGATGCCGCCTTTAA-3′, reverse 5′-CATTCCAGGCATCTGCGATGAG-3′; MMP9, forward 5′-GCCACTACTGTGCCTTTGAGTC-3′, reverse 5′-CCCTCAGAGAATCGCCAGTACT-3′; MMP13, forward 5′-CCTTGATGCCATTACCAGTCTCC-3′, reverse 5′-AAACAGCTCCGCATCAACCTGC-3′; NFATc1, forward 5′-GGTGCCTTTTGCGAGCAGTATC-3′, reverse 5′-CGTATGGACCAGAATGTGACGG-3′; Trap, forward 5′-GCGACCATTGTTAGCCACATACG-3′, reverse 5′-CGTTGATGTCGCACAGAGGGAT-3′; Ctr, forward 5′-CTGGGATGGCTGGATGTG-3′, reverse 5′-TGCTGTCAGGGTGTCTAAAC-3′; Ctsk, forward 5′-AGCAGAACGGAGGCATTGACTC-3′, reverse 5′-CCCTCTGCATTTAGCTGCCTTTG-3′; DC-STAMP, forward 5′-TTTGCCGCTGTGGACTATCTGC-3′, reverse 5′-GCAGAATCATGGACGACTCCTTG-3′; VATPase D2, forward 5′-ACGGTGATGTCACAGCAGACGT-3′, reverse 5′-CTCTGGATAGAGCCTGCCGCA-3′.

### ELISA

We collected supernatants from different cells after specific treatment. Then MMPs and pro-inflammatory cytokines were detected with ELISA kits (Bangyi, Shanghai, China) on the basis of the prepared standard curve.

### In vitro osteoclast differentiation assay

BMMs were inoculated into 96-well plates at a density of 8 × 10^3^ cells/well and adhered to the wall for 2 days. Various TOD contents (0, 5, 10, and 20 µM) were dissolved in the medium to test its effect on osteoclast differentiation. The osteoclastogenic medium was altered every two days until the fifth day. 4% PFA was used to fix the cultured BMMs, and the formation of osteoclasts was observed by Trap staining (Solarbio, Beijing, China).

### Bone resorption pit assay

Round calf bone slices measuring 8 mm (STX0012A, Thousand Sunrise, China) were autoclaved and placed in 96-well plates. The BMMs were inoculated on calf bone slices at a density of 8 × 10^3^ cells/well, and osteoclast differentiation was induced in α-MEM containing medium supplemented with 25 ng/ml M-CSF and 50 ng/ml RANKL. Different TOD contents (0, 5, 10, and 20 µM) were dissolved in the medium to test their effects on osteoclast differentiation. After 5 days, calf bone slices were removed, and cells on the surfaces were brushed off.

### Nuclear and cytoplasmic extraction

After treatment of the SW1353 and BMMs cells with TOD under different conditions, we extracted nuclear and cytoplasmic proteins for observing the expression of P65 and P-P65. Nuclear and cytoplasmic proteins were extracted applying nuclear and cytoplasmic protein separation kits under the manufacturer’s instructions.

### Immunofluorescence staining

Treated cells were immobilised with 4% PFA at room temperature for 40 min and incubated employing 1% Triton for 15 min for membranes penetrations. Then, cells were then blocked with 0.2% BSA for 1 h and cultured adopting a primary antibody at 4 °C overnight. After staining the nuclei employing DAPI for 10 min, the cells were rinsed three times applying sterile PBS and observed under a fluorescence microscope (Olympus FluoViewTM FV1000, Tokyo, Japan).

### Construction of Mouse OA models

To study the therapeutic effects of TOD on the mouse knee joint, we surgically constructed a mouse OA model through anterior cruciate ligament transection (ACLT) of the knee joint. Twenty 8-week-old male C57BL/6 mice from the Animal Center of the Chinese Academy of Sciences were raised at the Experimental Animal Department of Shanghai General Hospital. They were divided into four groups with five mice in each group: sham, ACLT + PBS, ACLT + Low TOD, and ACLT + High TOD groups. Specifically, in the ACLT group, mice were anesthetized through intraperitoneal injection of 4% chloral hydrate, and ACLT was performed using a needle in the left knee without skin incision. Anterior drawer detection was used for testing the effect of surgery. Mice of the sham group (n = 5) were not operated on. Mice of the ACLT + PBS group (n = 5) underwent ACLT, and PBS (30 mg/kg) was injected intraperitoneally. Mice of the ACLT + Low TOD underwent ACLT and we injected TOD (6 mg/kg) intraperitoneally. Mice of the ACLT + High TOD group underwent ACLT, and we injected TOD (12 mg/kg) intraperitoneally. All experiments were performed in accordance with prescribed animal welfare procedures, and this investigation was approved by the Ethics Committee of Shanghai General Hospital (#2021AW064).

### Histological assessments

Mice left knees were fixed in 4% PFA for 24 h at 6 weeks post-operation. All knees were decalcified adopting 10% EDTA for 1 month and embedded in paraffin. Subsequently, they were sliced into 4 μm sections and stained with H&E, Safranin O/Fast Green, and Toluidine Blue.

### TUNEL staining

Apoptotic cells in the articular cartilage were detected with terminal deoxynucleotide transferase dUTP staining under the manufacturer’s instructions. The quantity of TUNEL-positive cells was quantified.

### Statistical analysis

All data exhibited as the average ± SD of three independent experiments. SPSS software was used for data analysis. One-way ANOVA was employed for statistic analyses (^*,#^ indicates p < 0.05, ^**,##^ indicates p < 0.01, ^ns^ indicates not significant).

## Results

### TOD inhibits expression of inflammatory and catabolic mediators in LPS/IL-1β treated chondrocytes in vitro

**Fig. 1:**
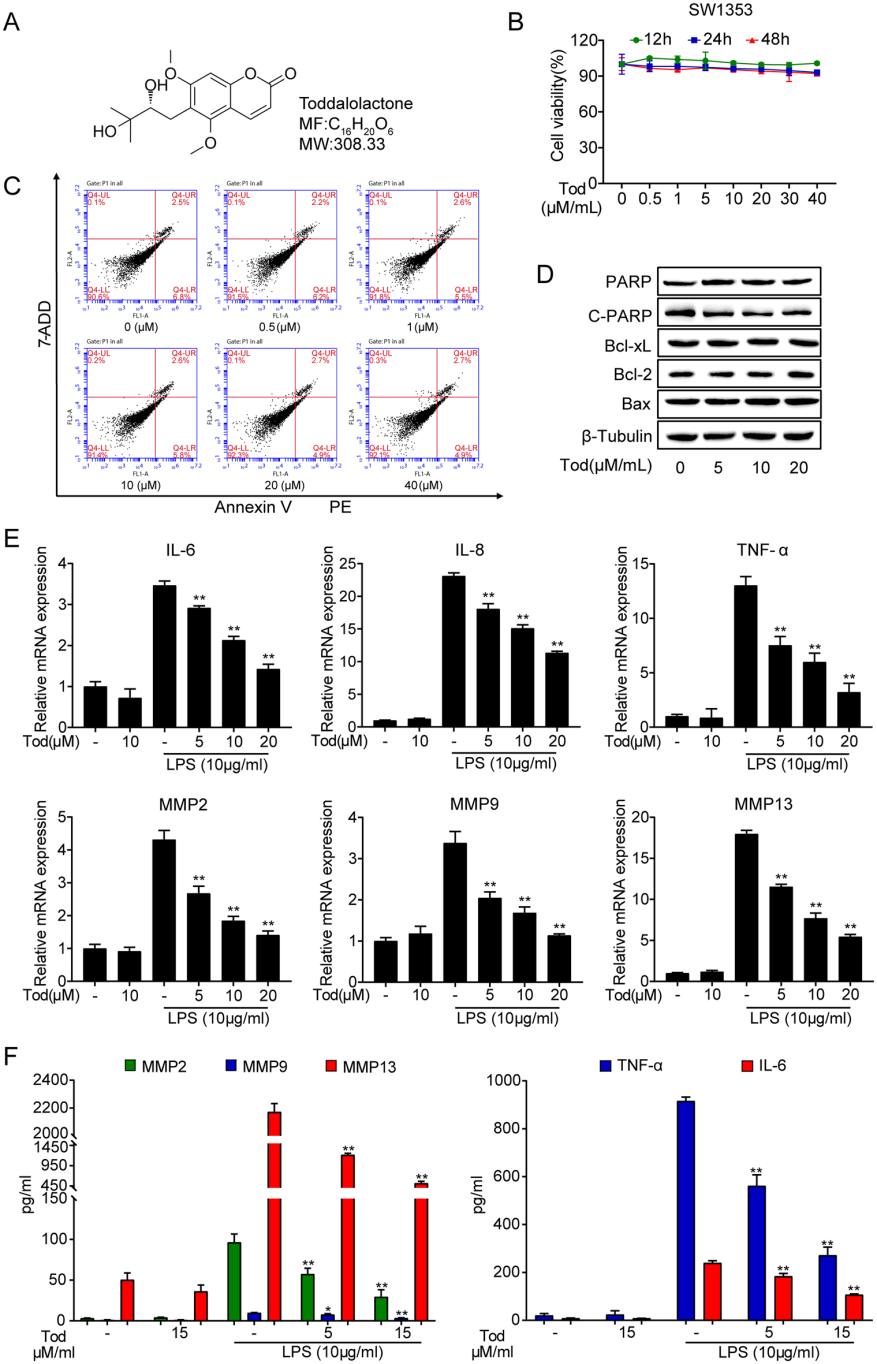
TOD inhibited the expression of inflammatory and catabolic mediators in LPS-induced chondrocytes. **A** Chemical structure of TOD. **B** The effect of TOD on the cell viability of SW1353 was evaluated by CCK-8. **C** Gradient concentrations (0, 0.5, 1, 5, 10, 20, 40 µM/ml) of TOD on the apoptosis level of SW1353 was measured by flow cytometry. **D** Western blotting performed after SW1353 cells were treated with gradient concentrations (0, 5, 10, and 20 µM/ml) of TOD; β-Tubulin was used as the loading control. **E** Pro-inflammatory cytokines, including IL-6, IL-8, TNF-α, MMP2, MMP9, and MMP13 were measured by RT-PCR. **F** ELISA was used to measure the expression level of MMP2, MMP9, MMP13, IL-6, and TNF-α. Data represent means ± SD of triplicate independent experiments. ^*, #^ indicates p < 0.05, ^**, ##^ indicates p < 0.01, ^ns^ indicates not significant

Figure 1A shows the molecular structure of TOD. For investigating the toxic impact of TOD on chondrocytes, we used the CCK8 assay kit, western blot assay and flow cytometry analysis to detect the effect of gradient concentrations (0, 0.5 1, 5, 10, 20, and 40 µM) of TOD on chondrocytes. Figure 1B–D show that no significant cytotoxicity of TOD on chondrocytes was observed less than 40 µM/ml. The percentages of apoptotic cells in the flow cytometry analysis and the western blot bands were quantified (Additional file 1: Fig. S1A and S1B). Our next experiment used TOD concentrations in the same range. LPS is commonly used to induce in vitro arthritic conditions [34]. Results of the RT-PCR analysis revealed that LPS stimulation significantly increased chondrocytes pro-inflammatory cytokine and MMP levels, while TOD significantly reduced this LPS-induced increase (Fig. 1E). ELISA revealed that TOD reduced the protein levels of MMP2, MMP9, MMP13, IL-6, and TNF-α secreted by chondrocytes after treatment with LPS (Fig. 1F). IL-1 β is another commonly used stimulator in inflammatory chondrocyte models [35–37]. To further verify the anti-inflammatory effect of TOD, we stimulated chondrocytes with IL-1β (10 ng/ml). The PCR and ELISA results were consistent with results from the LPS-stimulated chondrocyte model (Additional file 1: Fig. S2). These findings demonstrate that TOD inhibits the expression of inflammatory and catabolic mediators in LPS-treated chondrocytes.

### TOD inhibits RANKL-induced osteoclastogenesis, formation of F-actin ring, and bone resorption in vitro

**Fig. 2:**
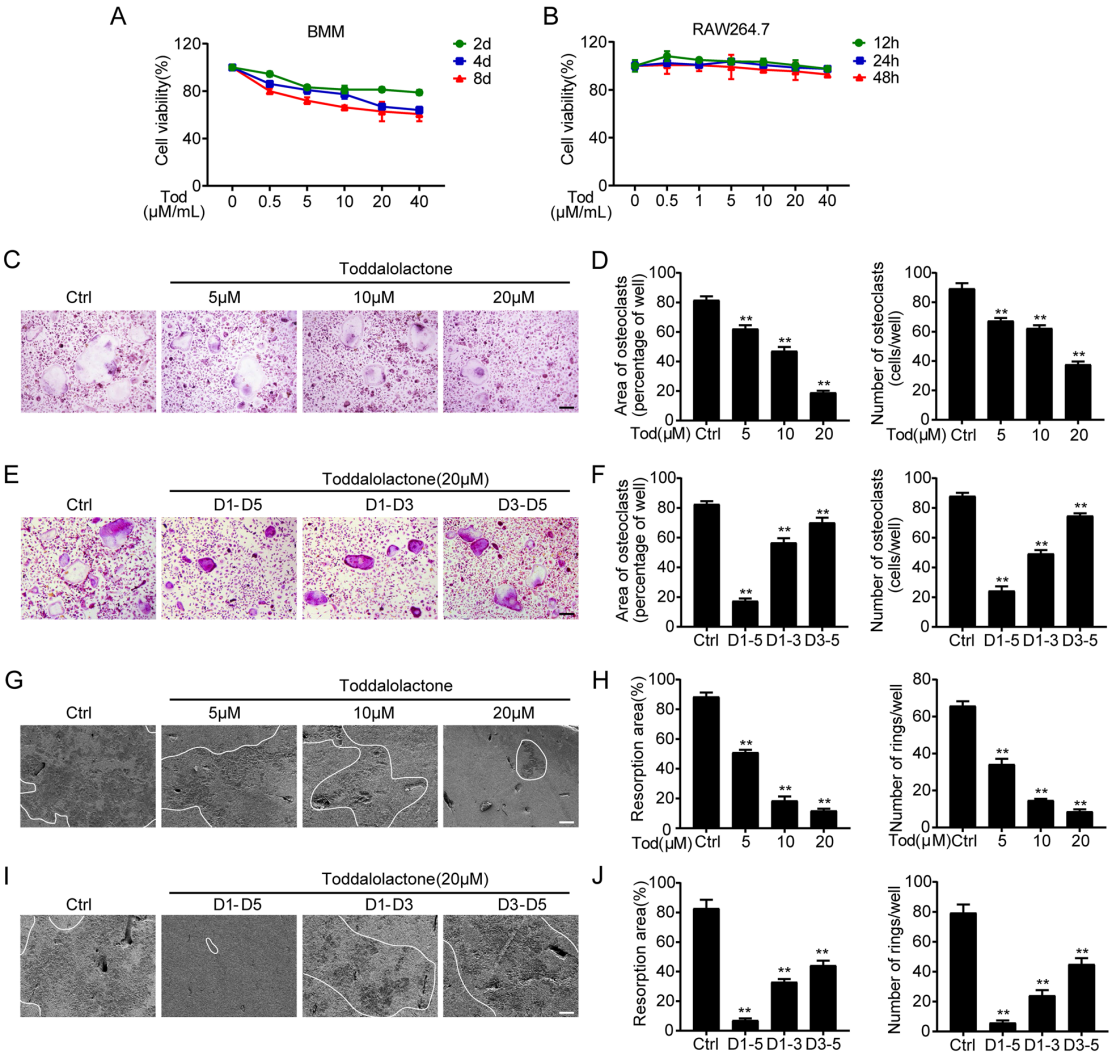
TOD inhibits RANKL-induced osteoclast differentiation and bone resorption without cytotoxicity in vitro. **A** The cell viability of BMMs exposed to TOD was measured by CCK-8. **B** The cell viability of RAW264.7 cells exposed to TOD was assessed by CCK-8. **C** BMMs were cultured with M-CSF (25 ng/ml), RANKL (50 ng/ml), and the indicated concentrations (0, 5, 10, and 20 µM) of TOD for 5 days. The effect of TOD on BMMs differentiation was detected using TRAP staining. Scale bar: 100 μm. **D** The area and number of osteoclasts were quantified per well. **E** BMMs were cultured with M-CSF (25 ng/ml) and RANKL (50 ng/ml) for 5 days, and TOD (20 µM) was added at different stages during osteoclast differentiation. The effect of TOD on BMMs differentiation was detected using TRAP staining. Scale bar: 100 μm. **F** The area and number of osteoclasts were quantified per well. **G** BMMs were cultured with M-CSF (25 ng/ml), RANKL (50 ng/ml), and the indicated concentrations (0, 5, 10, and 20 µM) of TOD for 5 days. Scanning electron microscopy was used to observe bone resorption pits. Scale bar: 100 μm. **H** The resorption area and number of rings were quantified. **I** BMMs were cultured with M-CSF (25 ng/ml) and RANKL (50 ng/ml) for 5 days, and TOD (20 µM) was added at different stages during osteoclast differentiation. Scanning electron microscopy was used to observe bone resorption pits. Scale bar: 100 μm. **J** The resorption area and number of rings were quantified. Data represent means ± SD of triplicate independent experiments. ^*, #^ indicates p < 0.05, ^**,##^ indicates p < 0.01, ^ns^ indicates not significant

**Fig. 3:**
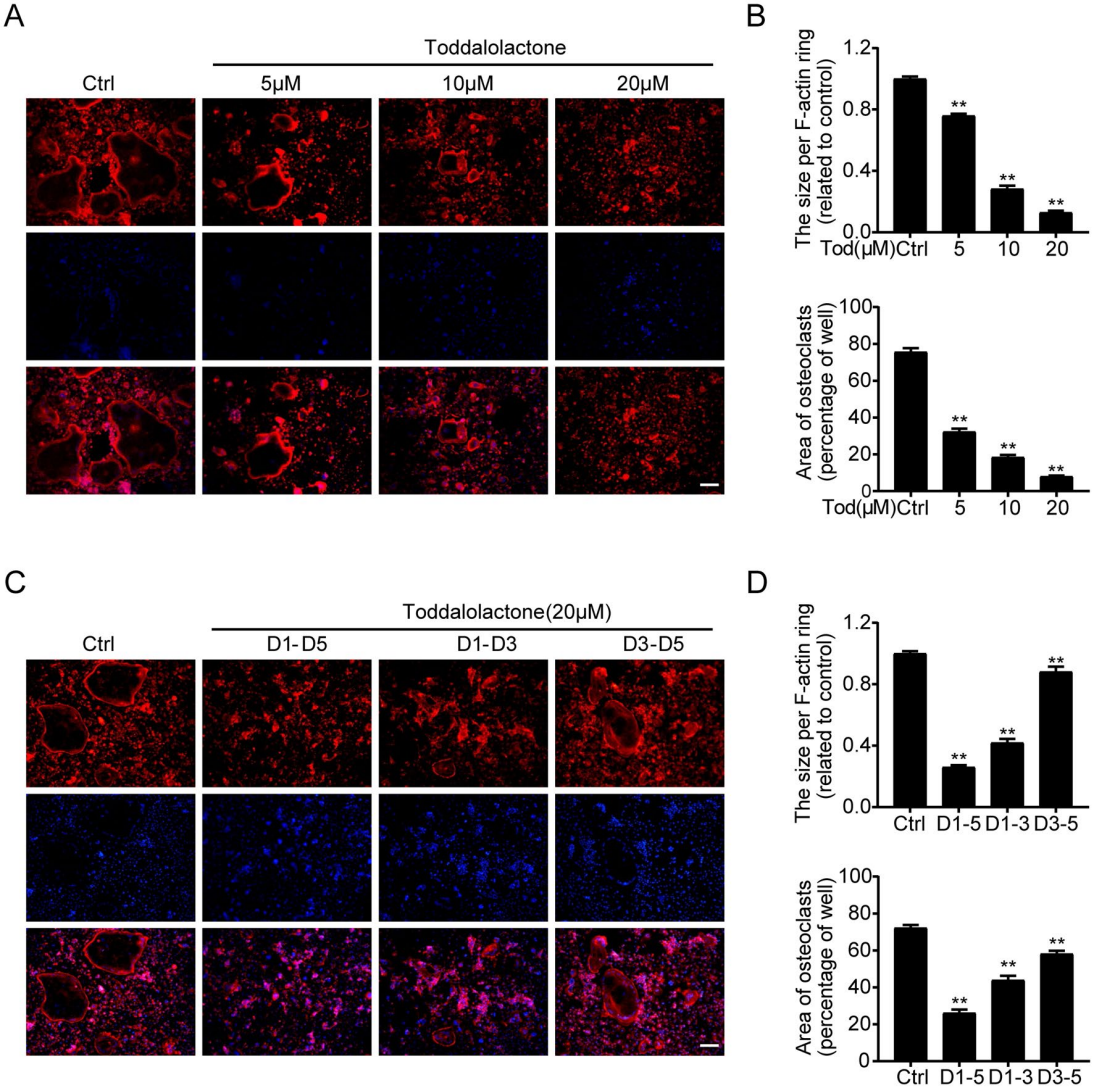
TOD inhibits the formation of F-actin ring. **A** BMMs were cultured with M-CSF (25 ng/ml), RANKL (50 ng/ml), and the indicated concentrations (0, 5, 10, and 20 µM) of TOD for 5 days. After differentiation, BMMs were fixed and stained for F-actin ring. Scale bar: 100 μm. **B** The size per F-actin ring and area of osteoclasts were quantified. **C** BMMs were cultured with M-CSF (25 ng/ml) and RANKL (50 ng/ml) for 5 days, and TOD (20 µM) was added at different stages during osteoclast differentiation. After differentiation, BMMs were fixed and stained for F-actin ring. Scale bar: 100 μm. **D** The size per F-actin ring and area of osteoclasts were quantified. Data represent means ± SD of triplicate independent experiments. ^*, #^ indicates p < 0.05, ^**, ##^ indicates p < 0.01, ^ns^ indicates not significant

The results of our CCK8 assay kit present that TOD exhibited no cytotoxic effects in either BMMs or RAW264.7 cells up to a concentration of 20 µM (Fig. 2A and B). Therefore, for the following experiments, no more than 20 µM of TOD doses was used. An in vitro osteoclastogenesis model is required for exploring how TOD affects osteoclastogenesis [38]. When the cells were stimulated with various TOD contents (0, 5, 10, and 20 µM/ml) for 5 days, the number of osteoclasts gradually decreased (Fig. 2C). Quantification of the area occupied by the osteoclasts and number of osteoclasts (Fig. 2D) revealed the same pattern. To investigate the stage of osteoclast formation, we supplemented 20 µM TOD to the osteoclastogenic medium at three different phases: d1 to d3 (early phase), d3 to d5 (late phase), and d1 to d5 (all phases). Compared with late phase intervention, early TOD intervention significantly inhibited osteoclastogenesis (Fig. 2E and F). We next used bone resorption assays to test osteoclast formation. Then the same amount of BMMs were inoculated onto the calf bone slices. As increasing TOD concentrations significantly inhibited osteoclastogenesis (Fig. 2G and H). The same trends are presented in Fig. 2E–J. Immunofluorescence staining showed that increasing TOD concentrations gradually suppressed actin ring formation (Fig. 3A and B). Additionally, the inhibitory effect was concentration-dependent and stronger when TOD was added in the early phase (Fig. 3C and D). These results suggest that TOD significantly inhibited osteoclastogenesis, especially in the early phase.

### TOD inhibits RANKL-induced osteoclast-related gene expression in vitro

**Fig. 4:**
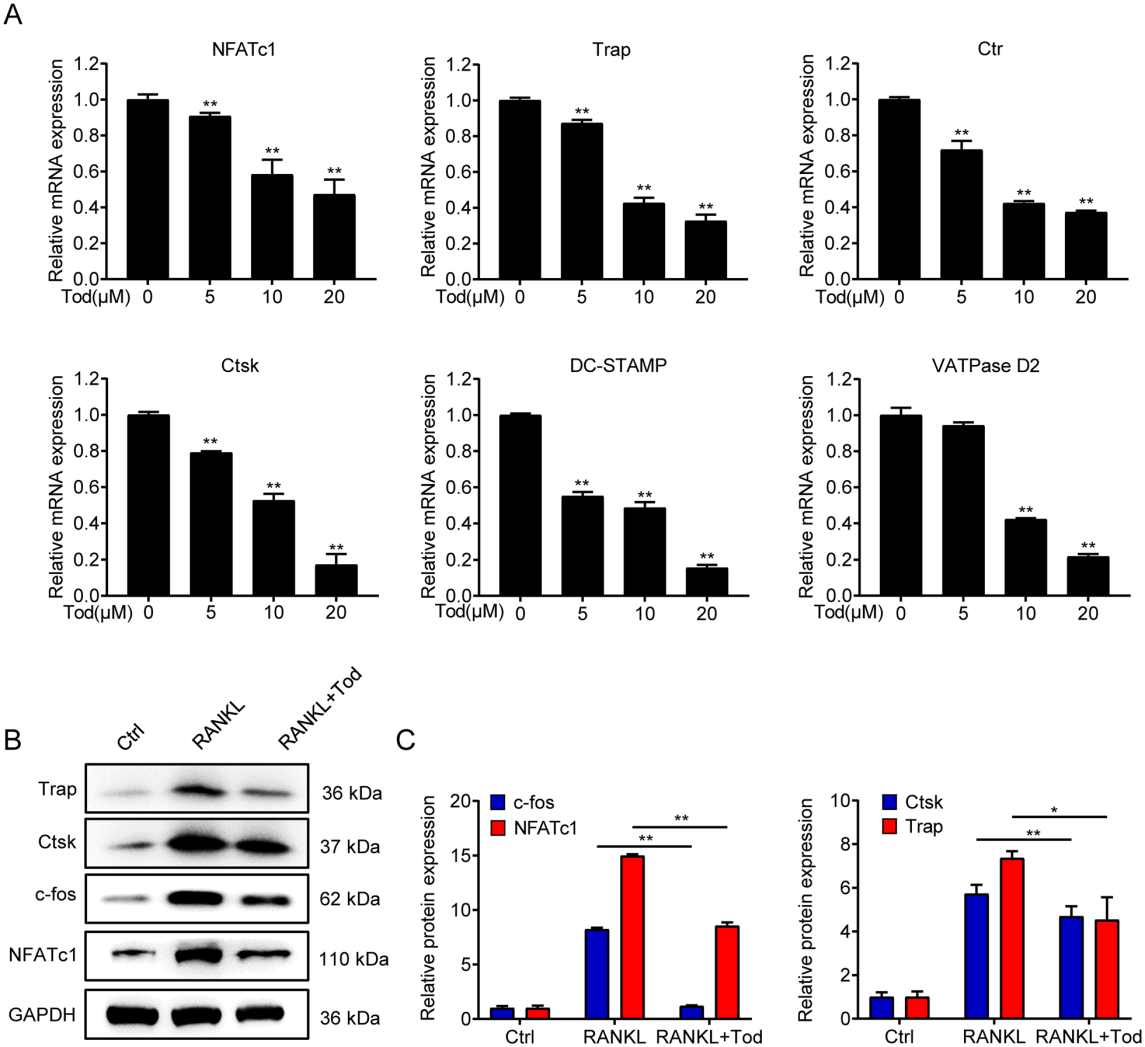
TOD suppresses RANKL-induced gene expression during osteoclastogenesis in vitro. **A** mRNA expression of osteoclast marker genes (NFATc1, Trap, Ctr, Ctsk, DC-STAMP, and VATPase D2) were detected by RT-PCR. **B** Western blotting performed that TOD suppressed the activation of osteoclast related protein (Trap, Ctsk, c-Fos, and NFATc1); GAPDH was used as the loading control. **C** ImageJ software was used to quantify the density of the western blot bands shown in **B**. Data represent means ± SD of triplicate independent experiments. ^*, #^ indicates p < 0.05, ^**, ##^ indicates p < 0.01, ^ns^ indicates not significant

Many studies have confirmed that blocking the RANKL/RANK interaction induced cascades can significantly inhibit osteoclast formation [21]. For studying the mechanism of TOD in inhibiting osteoclastogenesis, mRNA expression of osteoclast marker genes (NFATc1, Trap, Ctr, Ctsk, DC-STAMP, and VATPase D2) were measured through RT-PCR. We found that TOD reduced the expression of osteoclast formation marker genes in a dose-dependent manner (Fig. 4A). Outcomes of western blots analysis exhibited that TOD significantly suppressed the expression of Trap, Ctsk, Ctr, c-Fos and NFATc1 (Fig. 4B, C and Additional file 1: Fig. S3). These findings indicate that TOD suppressed RANKL-induced osteoclast-related gene expression in vitro.

### TOD inhibits activation of NF-κB and MAPK signalling pathways

**Fig. 5:**
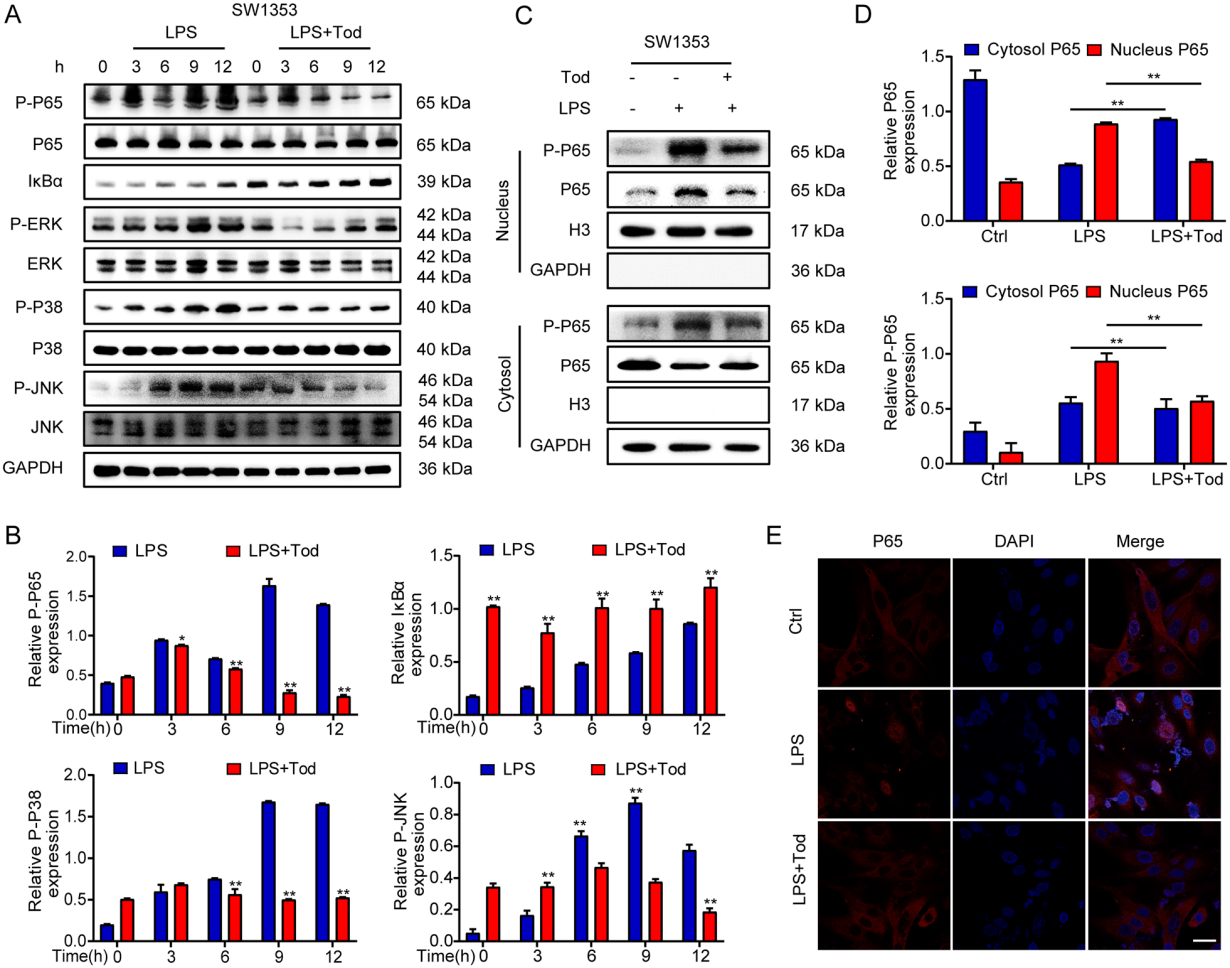
TOD inhibits the activation of the NF-κB and MAPK signalling pathways in SW1353 cells. **A** Western blotting performed the activity of NF-κB and MAPK signalling pathways in SW1353 cells; GAPDH was used as the loading control. **B** ImageJ software was used to quantify the density of the western blot bands (P-P65, IκBα, P-P38, and P-JNK) shown in A. **C** Western blots performed the protein expression of P65 in the nucleus and cytoplasm in SW1353 cells. **D** ImageJ software was used to quantify the density of the western blot bands in **C**. **E** Immunofluorescence was used to determine nuclear translocation of p65 in SW1353 cells. Scale bar: 100 μm. Data represent means ± SD of triplicate independent experiments. ^*, #^ indicates p < 0.05, ^**, ##^ indicates p < 0.01, ^ns^ indicates not significant

**Fig. 6:**
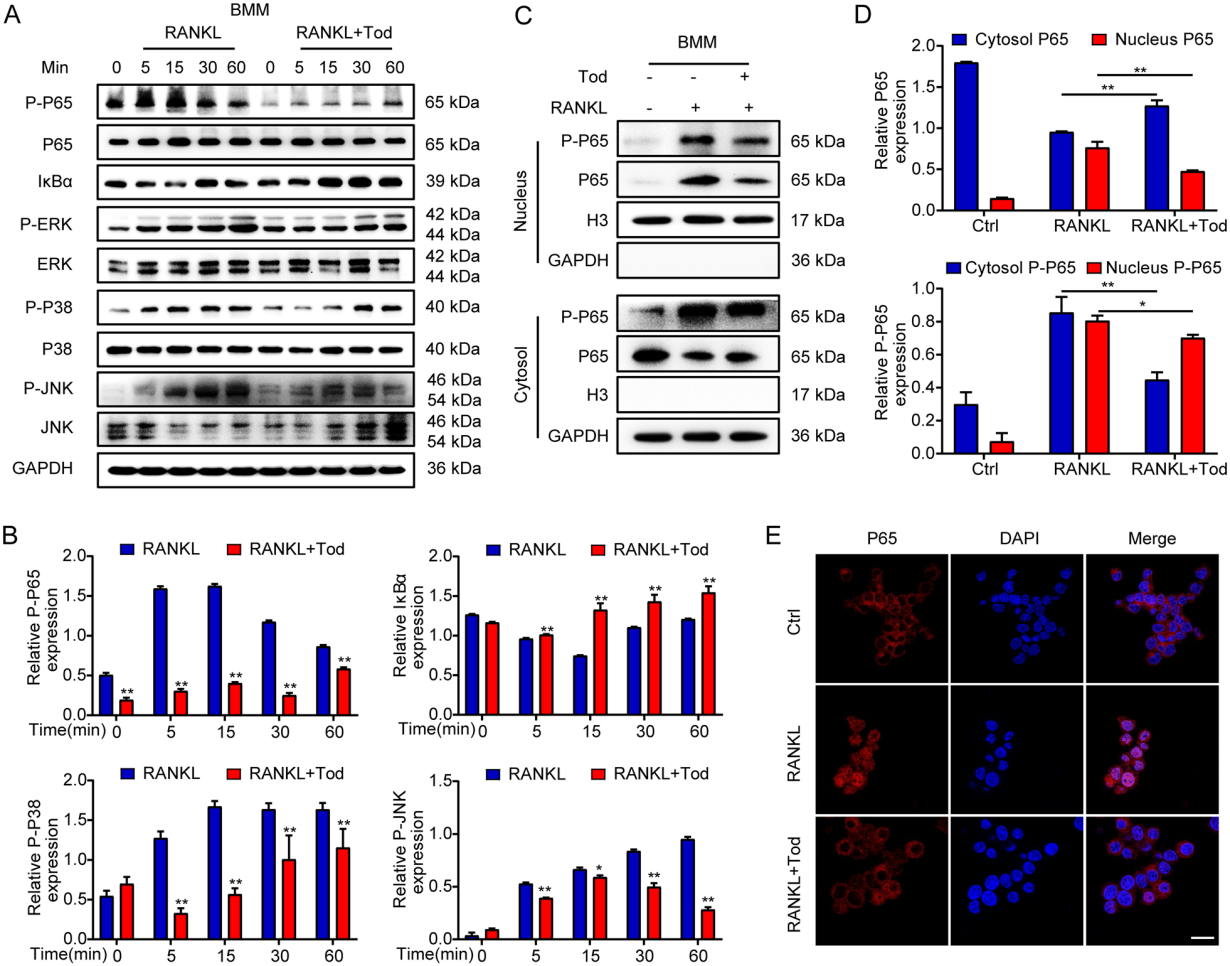
TOD attenuates the activation of the NF-κB and MAPK signalling pathways in BMMs. **A** Western blotting performed the activity of NF-κB and MAPK signalling pathways in RAW264.7 cells; GAPDH was used as the loading control. **B** ImageJ software was used to quantify the density of the western blot bands (P-P65, IκBα, P-P38, and P-JNK) shown in **A**. **C** Western blots performed the protein expression of P65 in the nucleus and cytoplasm in RAW264.7 cells. **D** ImageJ software was used to quantify the density of the western blot bands in **C**. **E** Immunofluorescence was used to determine nuclear translocation of p65 in RAW264.7 cells. Scale bar: 100 μm. Data represent means ± SD of triplicate independent experiments. ^*, #^ indicates p < 0.05, ^**, ##^ indicates p < 0.01, ^ns^ indicates not significant

As a major metabolic pathway in OA, NF-κB signalling pathway is involved in LPS-induced inflammation and RANKL-induced osteoclast formation. After stimulation, the activated NF-κB molecule triggers the expression of a series of genes that induce the destruction of articular cartilage and active osteoclast formation. The NF-κB signalling pathway is considered as an essential regulator of cartilage destruction and bone remodeling in OA [22, 39, 40]. To investigate the mechanism of TOD on chondrocyte inflammation, we stimulated SW1353 cells with LPS or LPS+TOD for various time periods (0, 3, 6, 9, and 12 h). We also investigated the same interaction in terms of osteoclast formation through treating BMMs with RANKL or RANKL+TOD for various durations (0, 5, 15, 30, and 60 min). Western blotting was adopted for detecting the expression of the NF-κB signalling pathway. Unlike in the LPS group or the RANKL group, TOD treatment greatly inhibited the degradation of IκBα and lessened the expression of P-P65, which is the key transcription factor associated with NF-κB signalling pathway (Figs. 5A and 6A). Figures 5B and 6B show the quantification of the western blot banks. Additionally, we then extracted nuclear and cytoplasmic proteins to detect the translocation of p65. TOD suppressed the translocation of P65 into the nucleus (Figs. 5C, D and  6C, D). Immunofluorescence staining (Figs. 5E and 6E) revealed that the nuclear translocation of p65 was reduced after TOD treatment. The MAPK signalling pathway has been reported to be significantly associated with OA cartilage damage and bone remodeling [41, 42]. In this investigation, TOD obviously inhibited the JNK, ERK, and P38 phosphorylation in SW1353 and BMMs (Figs. 5A and  6A). Our data indicate both NF-κB and MAPK signalling pathways were suppressed, which suggested that TOD may restrain the destruction of articular cartilage and osteoclastogenesis by inhibiting the activation of the NF-κB and MAPK signalling pathways.

### TOD prevents against ACLT-induced cartilage destruction and inhibits osteoclastogenesis in vivo

**Fig. 7:**
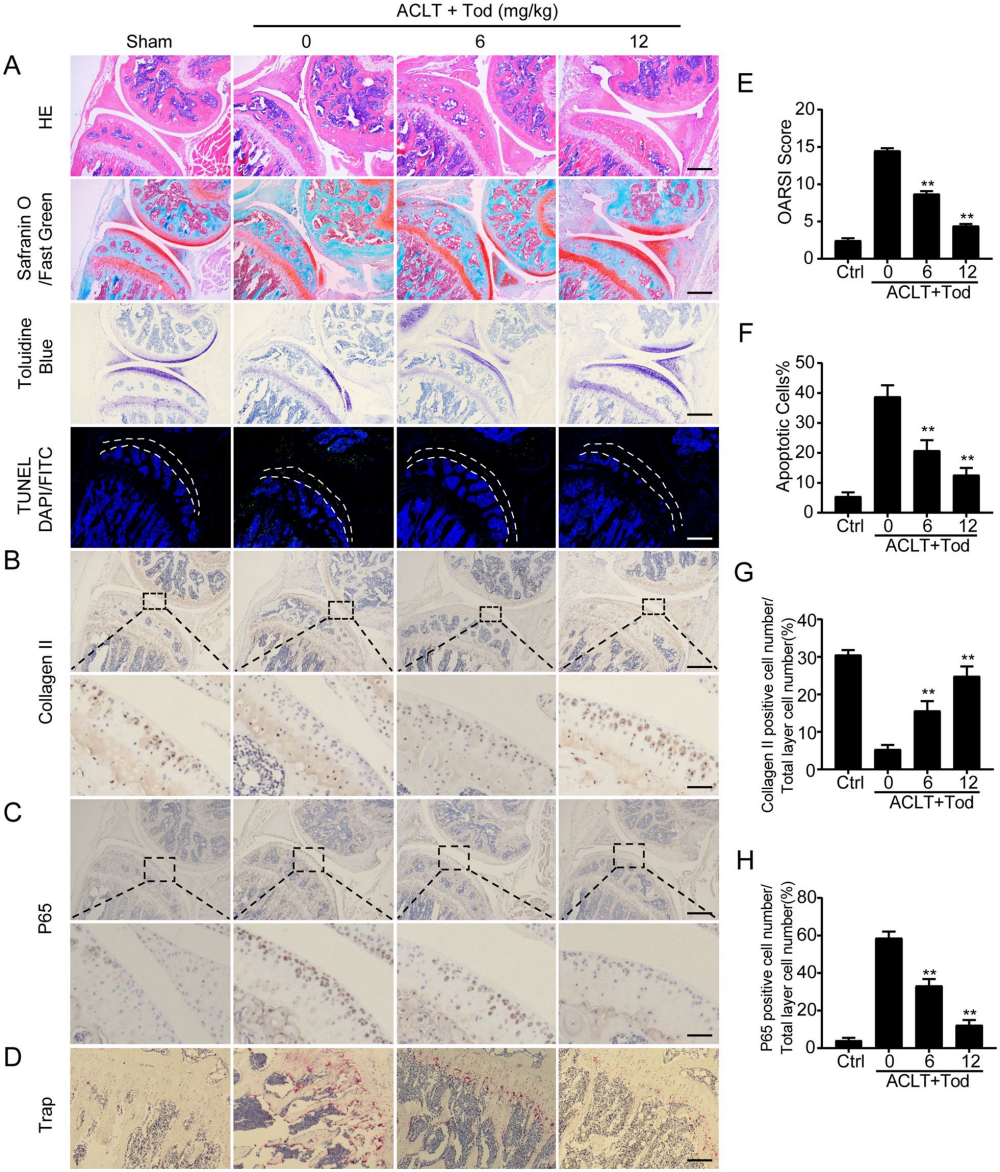
TOD protects against ACLT-induced cartilage destruction and inhibits osteoclastogenesis in vivo. **A** H&E staining, Safranin O/Fast Green staining, Toluidine Blue, and TUNEL DAPI/FITC staining were performed respectively. Scale bar: 100 μm. **B** Immunohistochemical analysis of Collagen II. Scale bar: 100 μm (upper); 10 μm (lower). **C** Immunohistochemical analysis of P65. Scale bar: 100 μm (upper); 10 μm (lower). **D** TRAP staining of the osteoclasts in the subchondral plate. Scale bar: 200 μm. **E** The OARSI scores of different groups were assessed. **F** The percentage of apoptotic chondrocytes in cartilage was calculated. **G** Quantitative analysis of the immunohistochemical staining of Collagen II. **H** Quantitative analysis of the immunohistochemical staining of P65. Data represent means ± SD of triplicate independent experiments. ^*, #^ indicates p < 0.05, ^**, ##^ indicates p < 0.01, ^ns^ indicates not significant

For further studying the protective impact of TOD on OA in vivo, we first constructed a mouse OA model through ACLT of the knee joint, which is a classical model for the study of OA. Based on the previous description, the mice were classified into four groups with five C57BL/6J mice in each group. H&E staining and Safranin O/Fast Green staining (Fig. 7A) revealed cartilage erosion in the ACLT + PBS group that was attenuated with ACLT + TOD treatment. We then quantified the structural alterations in tibial plateau cartilage using the OARSI scoring standard (Fig. 7E). Each sample in the treatment group was independently evaluated by two experienced experimentalists blinded to the study conditions. The two observers also agreed on the results. We next used TUNEL DAPI/FITC staining for measuring chondrocyte apoptosis. Compared with the Sham mice, TUNEL-positive cells increased significantly in the ACLT+PBS group and decreased in the ACLT + TOD group (Fig. 7A). The number of apoptotic cells was counted (Fig. 7F). Immunohistochemical staining revealed that ACLT + PBS decreased Collagen II expression in cartilage compared with the sham group, and TOD treatment recovered this decrease (Fig. 7B and G). The ACLT + PBS treatment also increased the percentage of p65-positive cells, a change that TOD again reversed (Fig. 7C and H). However, MAPK-positive cells did not differ significantly between the groups. Trap staining of the subchondral trabecular bone showed that Trap-positive cells increased significantly in the ACLT + PBS group along with osteoclast activity; these changes were attenuated in the ACLT + TOD group (Fig. 7D). Taken together, these data have confirmed that TOD prevents against ACLT-induced cartilage destruction and inhibits osteoclastogenesis in vivo.

### TOD prevents against ACLT-induced bone loss in vivo

**Fig. 8:**
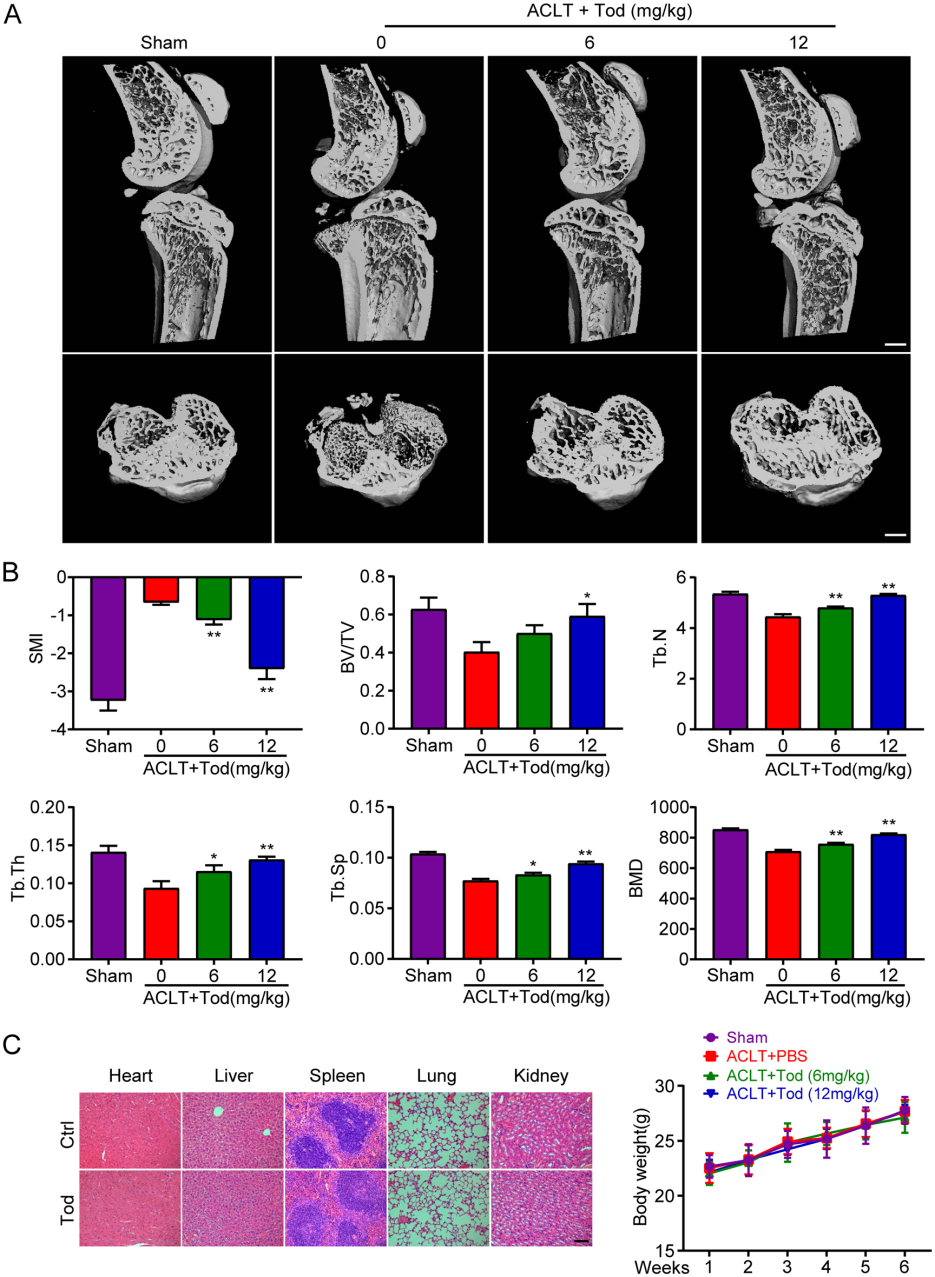
TOD protects against ACLT-induced bone loss in vivo. **A** 3D reconstruction micro-CT images were performed for each group. Scale bar: 2 mm. **B** Quantification of SMI, BV/TV, Tb.Th, Tb.Sp, Tb.N, and BMD. **C** H&E staining of important organs. Scale bar: 200 μm; Body weights were recorded once per week. Data represent means ± SD of triplicate independent experiments. ^*, #^ indicates p < 0.05, ^**,##^ indicates p < 0.01, ^ns^ indicates not significant

Subchondral bone resorption often occurs after ACLT in mice [43]. For evaluating how TOD therapeutically affects subchondral bone loss after ACLT in mice, we used 3D reconstruction micro-CT to examine the structure of the subchondral bone. Loss of subchondral bone mass was significant in the ACLT group, and intraperitoneal injection of TOD significantly inhibited bone resorption (Fig. 8A). SMI, BV/TV, BMD, Tb.N, Tb.Th, and Tb.Sp were detected on basis of the 3D reconstruction images (Fig. 8B). Furthermore, TOD had no adverse effects on key organs and body weight in mice (Fig. 8C). These outcomes imply that TOD is a potential therapeutic agent for OA.

## Discussion

Pathological changes in OA affects the whole joint, including focal and progressive hyaline cartilage loss, as well as subchondral bone changes, such as osteophytes development and increased bone capsule thickness [44]. Although these pathological processes may selectively affect individual articular tissues, there are close biological and physical interactions among them, and ultimately all components of the knee will interact with each other due to their close association [9]. At present, the purpose of non-surgical treatment for OA is mainly to relieve pain and delay progression. Therefore, actual curative drugs are necessary [45, 46]. In this study, we successfully demonstrated that TOD may be such drug.

Articular cartilage has no blood vessels or nerves and consists mainly of type II collagen and aggrecan, along with 70% water [47]. Chondrocytes are the only cells in cartilage and undergo important phenotypic changes during the development of OA. These changes include decreased secretion of type II Collagen and aggrecan, and increased secretion of inflammatory cytokines, MMPs, and ADATMs [48, 49]. In turn, the original homeostasis of cartilage tissue is disrupted, leading to OA. Here, we found that TOD suppressed the LPS/IL-1β-induced release of matrix-degrading enzymes and pro-inflammatory cytokines at doses that do cause toxic effects in chondrocytes, which can inhibit the low inflammatory state of chondrocytes and the transformation of chondrocytes into degraded phenotypes. These results suggested that in LPS/ IL-1β-induced chondrocytes in vitro, TOD suppressed the expression of inflammatory and catabolic mediators.

It is difficult to achieve good therapeutic effects in the treatment of OA because of chondrocytes characteristics. The subchondral bone plays a key role in OA progression [50]. During the development of OA, there are significant changes in the composition and structure of the subchondral bone, including raised cortical plate thickness, lessened subchondral cancellous bone mass, changes in architecture, and bone attrition [51]. These changes adversely affect the upper calcification and articular cartilage [52, 53]. Osteoclasts are key cells involved in subchondral bone remodeling. Our previous study showed that TOD dose-dependently inhibits RANKL-induced osteoclastogenesis, formation of F-actin ring, bone resorption, and inhibits osteoclast related genes in vitro. These data demonstrate that TOD is effective against bone loss.

The NF-κB and MAPK signalling pathways play important roles in chondrocyte inflammation and osteoclast formation [54, 55]. Studies have shown that inhibition of both pathways significantly improves the symptoms of OA and delays the progression of OA. Our results suggest that TOD inhibits NF-κB and MAPK signalling pathways by suppressing JNK, P65, P38, and ERK phosphorylation, which then inhibits LPS-induced chondrocyte inflammation and RANKL-induced osteoclast formation.

ACLT of the knee joint is a classical mouse OA model [56, 57]. In our study, we constructed the ACLT model that has similar pathological characteristics as human OA. Intraperitoneal injection of TOD significantly delayed the erosion of articular cartilage, reduced chondrocytes apoptosis, and inhibited osteoclast activity, thus delaying the progression of OA in the model mice. These results confirmed that TOD is a potential agent for treating OA. However, more research is necessary to determine whether TOD affects other targets that can delay OA progression. Examining inflammation specifically with chondrocytes and that with other immune cells will also be a focus in the future research.

## Conclusions

**Fig. 9:**
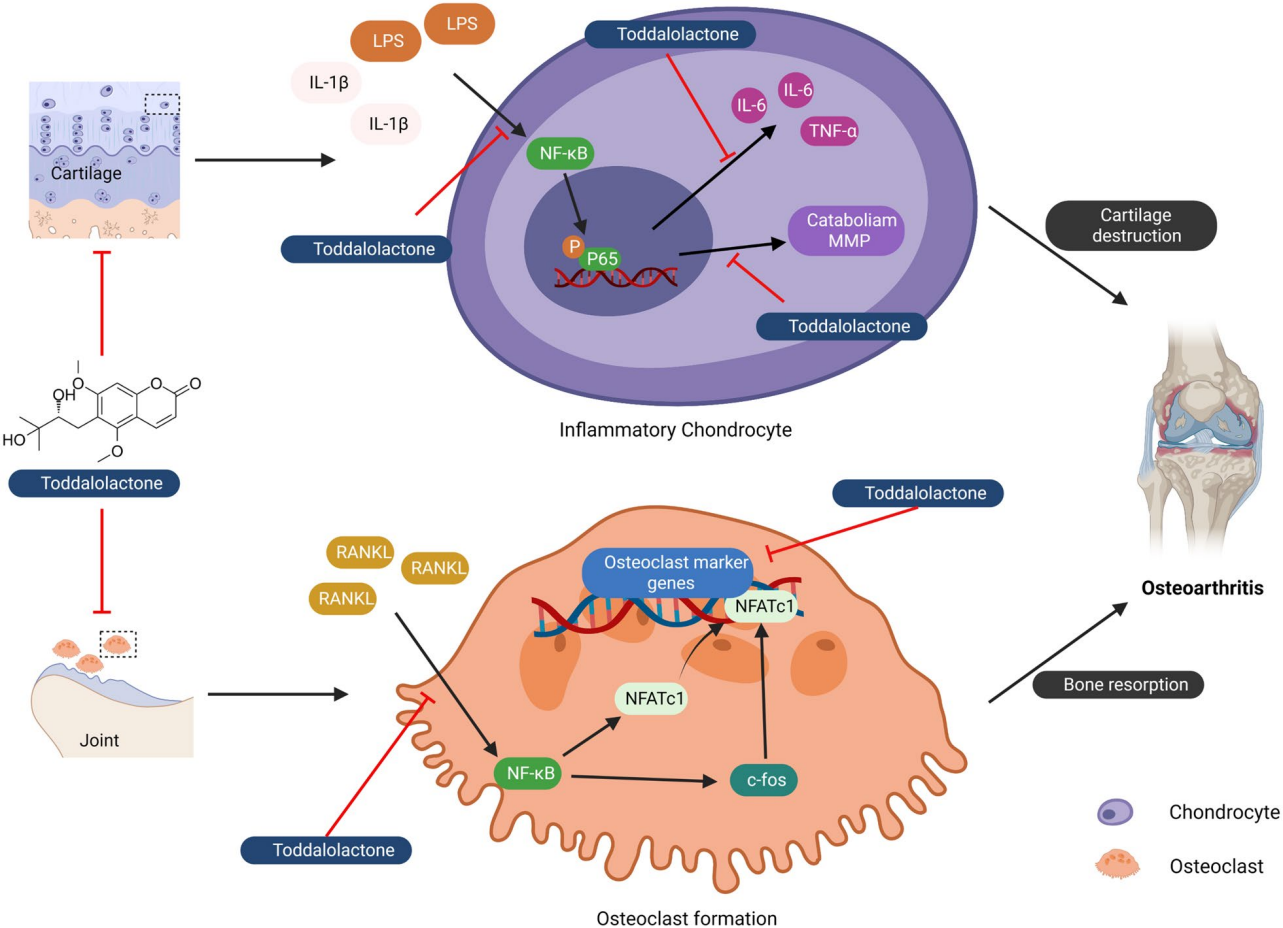
The mechanism of that TOD protects against osteoarthritis by ameliorating chondrocyte inflammation and suppressing osteoclastogenesis

In summary, our results are the first to demonstrate that TOD, a natural compound extracted from *Toddalia asiatica* (L.) Lam., attenuates the progression of OA, specifically through inhibiting matrix-degrading enzymes and pro-inflammatory cytokines secreted by chondrocytes. Figure 9 showed the mechanism of that TOD protects against OA by ameliorating chondrocyte inflammation and suppressing osteoclastogenesis (created with BioRender.com). TOD suppressed osteoclastogenesis through the inhibition of the NF-κB and MAPK signalling pathways. Our in vivo experiment with a mouse model confirmed that TOD delayed the erosion of articular cartilage, reduced the apoptosis of chondrocyte, and inhibited the activity of osteoclasts, thus delaying OA progression. In conclusion, we provided clear empirical evidence that TOD can potentially have important applications as a novel therapeutic target for OA.

## Supplementary Information

**Additional file 1: Fig. S1.** The quantification of apoptotic cells and the density of the western blot bands in Fig. 1. **Fig. S2.** TOD inhibited the expression of inflammatory and catabolic mediators in IL-1β induced chondrocytes. **Fig. S3.** TOD suppressed Ctr expression during osteoclastogenesis in vitro.

## Data Availability

The data included in this investigation are available from the corresponding author.

## Abbreviations

OA: Osteoarthritis
TOD: Toddalolactone
LPS: Lipopolysaccharides
BMMs: Bone marrow derived macrophages
NF-κB: Nuclear factor-kappaB
MAPK: Mitogen-activated protein kinase
MMPs: Matrix metalloproteinases
ADAMTS: A disintegrin and metalloproteinase with thrombospondin motifs
DMSO: Dimethyl sulfoxide
M-CSF: Macrophage colony stimulating factor
RANKL: Receptor activator of nuclear factor-Kappa B
TRAF6: Tumor necrosis factor receptor-associated factor 6
NFATc1: Nuclear factor of activated T cells c1
CTSK: Cathepsin K
DC-STAMP: Dendritic cell-specific transmembrane protein
CTR: Calcitonin receptor
TRAP: Tartrate-resistant acid phosphate
BSA: Bovine serum albumin
M-CSF: Macrophage colony stimulating factor
ACLT: Anterior cruciate ligament transection
SMI: Structure model index
Tb. Th: Trabecular thickness
Tb.N: Trabecular number
BMD: Bone mineral density
BV/TV: Bone volume/total tissue volume
Tb.Sp: Trabecular separation
