# Supplementary material for: Toddalolactone protects against osteoarthritis by ameliorating chondrocyte inflammation and suppressing osteoclastogenesis
Source: Chin Med. 2022 Feb 5;17:18. doi: 10.1186/s13020-022-00576-w (PMC8817519; doi:10.1186/s13020-022-00576-w)
Supplement: Supplementary file 1 — Additional file 1: Fig. S1. The quantification of apoptotic cells and the density of the western blot bands in Fig. 1. Fig. S2. TOD inhibited the expression of inflammatory and catabolic mediators in IL-1β induced chondrocytes. Fig. S3. TOD suppressed Ctr expression during osteoclastogenesis in vitro. [file 13020_2022_576_MOESM1_ESM.docx]

*Supplementary Information*

Toddalolactone protects against osteoarthritis by ameliorating chondrocyte inflammation and suppressing osteoclastogenesis

Yiming Xu^1,2†^, Song Xue^3†^, Tian Zhang^4†^, Xinmeng Jin^1,2^, Cong Wang^1^, Haiming Lu^1^, Yiming Zhong^1,2^, Hongjie Chen^1^, Libo Zhu^1*^, Jinzhong Ma^1*^ and Weilin Sang^1*^

*Correspondence: [sangweilin001@163.com](mailto:sangweilin001@163.com); [majinzhong1963@sina.com](mailto:majinzhong1963@sina.com); [libozhu2018@163.com](mailto:libozhu2018@163.com)

^†^Yiming Xu, Song Xue and Tian Zhang contributed equally to this work

^1^Department of Orthopedics, Shanghai General Hospital, Shanghai Jiao Tong University School of Medicine, Shanghai, China

^2^Shanghai Bone Tumor Institution, Shanghai, China

^3^Department of Rheumatology and Immunology, Arthritis Research Institute, The First Affiliated Hospital of Anhui Medical University, Hefei, China

^4^Department of Orthopedics, The Affiliated Yueqing Hospital, Wenzhou Medical University, Wenzhou, China

**Methods**

**Western blotting**

After treatment under different conditions, total proteins were collected from the cells adopting ice-cold RIPA lysis buffer. BCA protein assay (Beyotime, China) were adopted for measuring relative protein concentrations. After separation on SDS-PAGE gels, the protein was transferred to the PVDF membrane, which were sealed with 5% skimmed milk for 2 hours. Then the membrane was incubated overnight with a first antibody at 4°C. Enhanced chemiluminescence was employed for visualizing the bands. The following antibodies were used: Ctr (ab11042, Abcam), and GAPDH (60004–1-lg, Proteintech).

**Real-time PCR analysis**

After a specific treatment, total mRNA was extracted from various cells with TRIZOL. These RNA was reversely transcribed into cDNA adopting PrimeScript RT Master Mix (TaKaRa, Dalian, China). Real-time PCR was performed with TB Green Premix Ex Tap on basis of the manufacturer’s instructions. The primers employed are listed below: IL-6, forward 5’-CAACCTGAACCTTCCAAAGATG-3’, reverse 5’-ACCTCAAACTCCAAAAGACCAG-3’; IL-8, forward 5’-GAGAGTGATTGAGAGTGGACCAC-3’, reverse 5’-CACAACCCTCTGCACCCAGTTT-3’; TNF-α, forward 5’-CACTTCGAAACCTGGGATTCAG-3’, reverse 5’-GGTCTCCAGATTCCAGATGTCAG-3’; MMP2， forward 5’-AGCGAGTGGATGCCGCCTTTAA-3’, reverse 5’-CATTCCAGGCATCTGCGATGAG-3’; MMP9， forward 5’-GCCACTACTGTGCCTTTGAGTC-3’, reverse 5’-CCCTCAGAGAATCGCCAGTACT-3’; MMP13， forward 5’-CCTTGATGCCATTACCAGTCTCC-3’, reverse 5’-AAACAGCTCCGCATCAACCTGC-3’.

**ELISA**

We collected supernatants from different cells after specific treatment. Then MMPs and pro-inflammatory cytokines were detected with ELISA kits (Bangyi, Shanghai, China) on the basis of the prepared standard curve.

**Results**


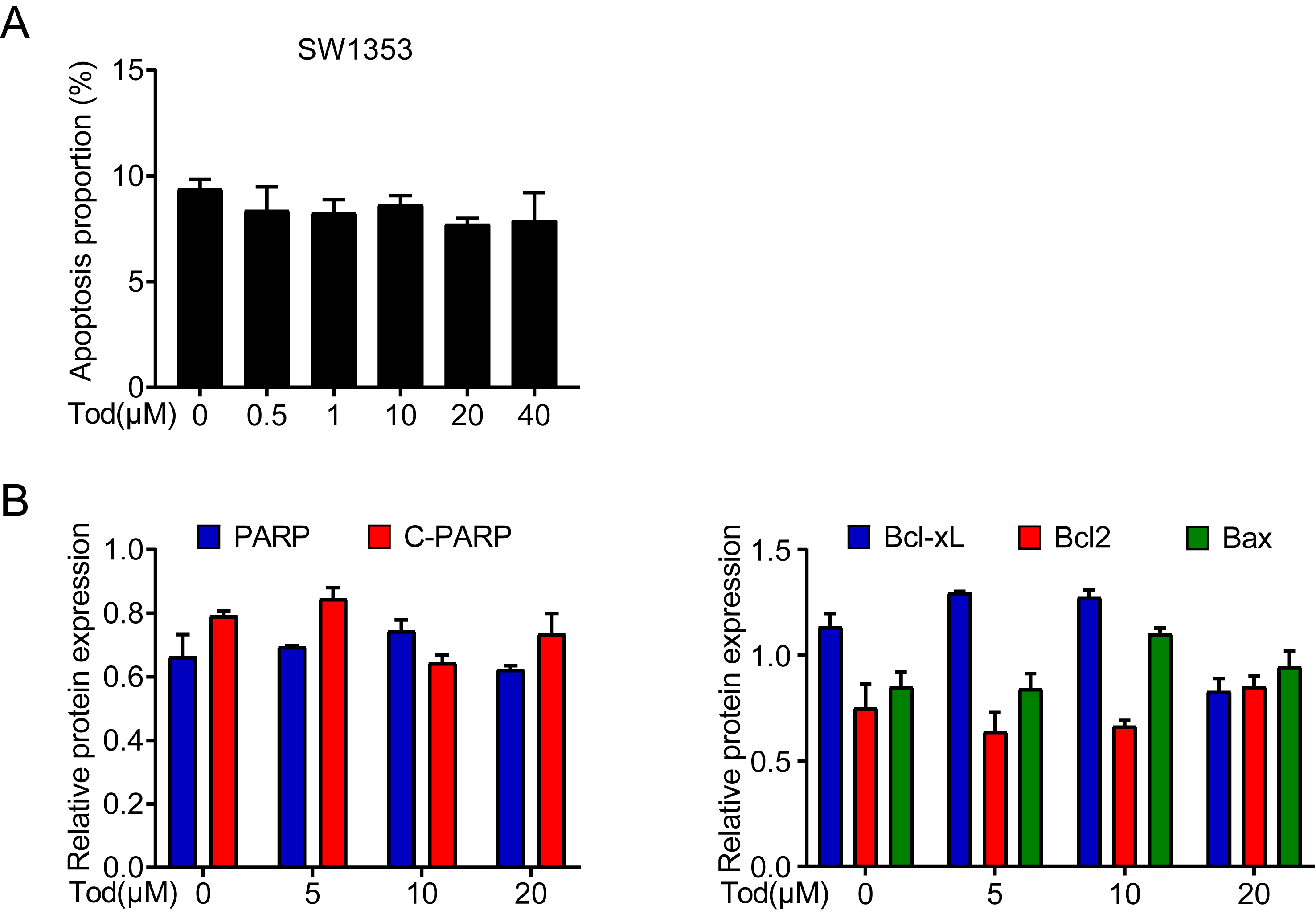


**Fig. S1** **A** The percentages of apoptotic cells in the indicated group in Fig. 1C are shown in the histogram. **B** ImageJ software was used to quantify the density of the western blot bands shown in Fig. 1D. Data represent means ± SD of triplicate independent experiments. ^*, #^indicates p < 0.05, ^**, ##^indicates p < 0.01, ^ns^ indicates not significant.


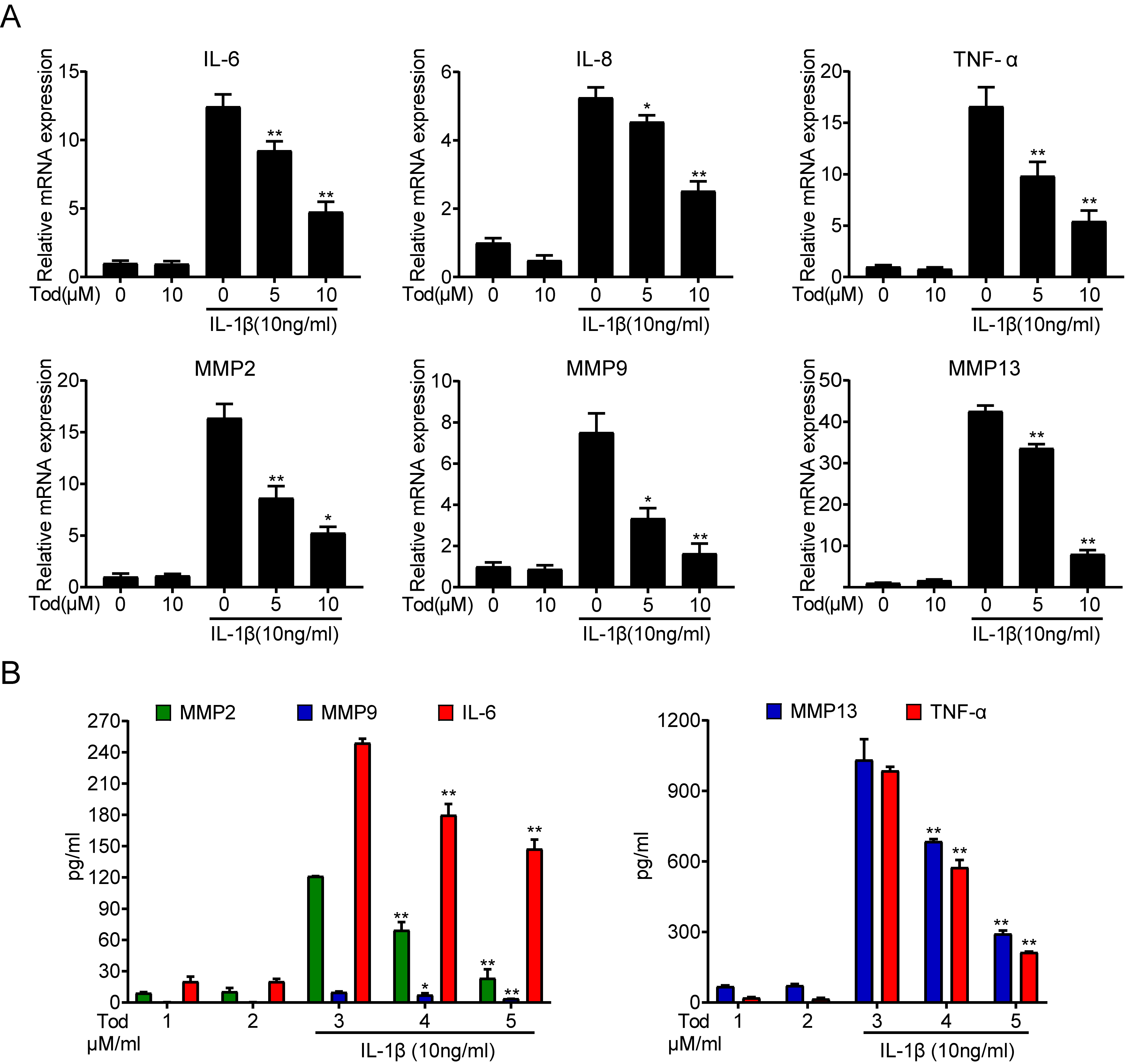


**Fig. S2** TOD inhibited the expression of inflammatory and catabolic mediators in IL-1β induced chondrocytes. **A** Pro-inflammatory cytokines, including IL-6, IL-8, TNF-α, MMP2, MMP9, and MMP13 were measured by RT-PCR. **B** ELISA was used to measure the expression level of MMP2, MMP9, MMP13, IL-6, and TNF-α. Data represent means ± SD of triplicate independent experiments. ^*, #^indicates p < 0.05, ^**, ##^indicates p < 0.01, ^ns^ indicates not significant.


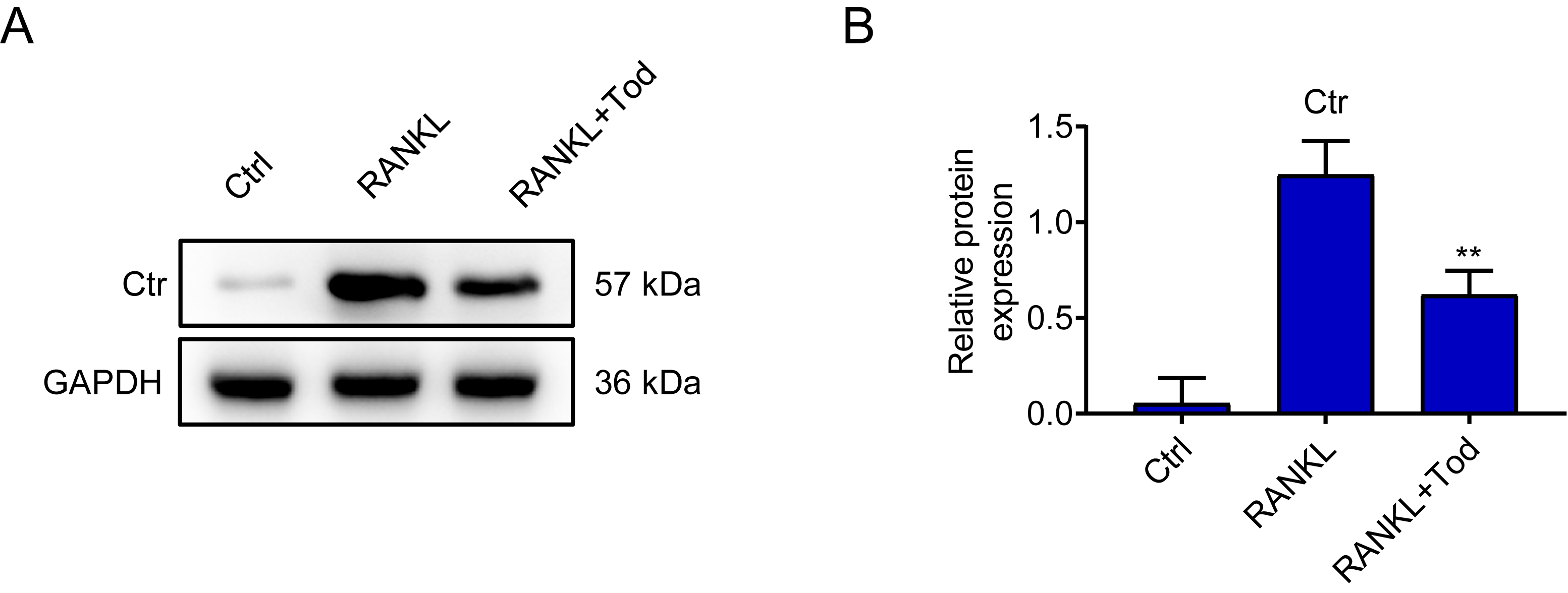


**Fig. S3** TOD suppresses Ctr expression during osteoclastogenesis *in vitro*. **A** Western blotting performed that TOD suppressed the activation of Ctr, which is the osteoclast related protein; GAPDH was used as the loading control. **B** ImageJ software was used to quantify the density of the western blot bands shown in A. Data represent means ± SD of triplicate independent experiments. ^*, #^indicates p < 0.05, ^**, ##^indicates p < 0.01, ^ns^ indicates not significant.
